# Interprofessional education and collaborative practice policies and law: an international review and reflective questions

**DOI:** 10.1186/s12960-020-00549-w

**Published:** 2021-01-07

**Authors:** Marie-Andrée Girard

**Affiliations:** 1 Anesthesiology and Pain Medicine Department, Faculty of Medicine, University of Montreal, Montreal, Canada; 2 Health Hub: Politics, Organizations and Law, Montreal, Canada; 3 Faculty of Law, University of Montreal, Montreal, Canada

**Keywords:** Interprofessional education and collaborative practice, Healthcare policy, Health system policy, Interprofessional policy, Health law

## Abstract

**Background:**

Healthcare is a complex sociolegal setting due to the number of policymakers, levels of governance and importance of policy interdependence. As a desirable care approach, collaborative practice (referred to as interprofessional education and collaborative practice (IPECP)) is influenced by this complex policy environment from the beginning of professionals’ education to their initiation of practice in healthcare settings.

**Main body:**

Although data are available on the influence of policy and law on IPECP, published articles have tended to focus on a single aspect of policy or law, leading to the development of an interesting but incomplete picture. Through the use of two conceptual models and real-world examples, this review article allows IPECP promoters to identify policy issues that must be addressed to foster IPECP. Using a global approach, this article aims to foster reflection among promoters and stakeholders of IPECP on the global policy and law environment that influences IPECP implementation.

**Conclusion:**

IPECP champions and stakeholders should be aware of the global policy and legal environment influencing the behaviors of healthcare workers to ensure the success of IPECP implementation.

## Background

Multiple pieces of evidence indicate that legal and organizational structures impact the lives and practice of health professionals [1–13]. However, empirical evidence on the actual influence of policy interventions is scarce [8, 9, 14, 15]. This paper aims to provide a review of the current literature as well as some orientation questions that researchers or key stakeholders wanting to promote interprofessional education and collaborative practice (IPECP) in their policies and systems must ask themselves.

### Conceptual model applicable to IPECP policy design

IPECP, as put forward by the WHO and the international organization Interprofessional. Global, is a comprehensive approach to collaborative care: from interprofessional education aimed at producing a collaboration-ready healthcare workforce to interprofessional collaborative practice and care at the patient bedside [16]. Two main models for conceptualizing the importance of policy and law for IPECP implementation and promotion are available in the literature.

The model of Mulvale et al. [2] allows the conceptualization of the intricate relations among micro-, meso-, and macro-level aspects. This model is organic, meaning that it relates one level to another through quasiphysiological linkages, with the model output being the resulting (individual-level) behaviors of the practicing professional. Its focus is on one organizational unit, meaning that it sets aside the tentacular nature of education and labor. Health providers on one team or in one facility may have backgrounds with different teaching structures, have different previous work experience, or come from different countries. From a policy perspective, the Mulvale model applies to one facility, one department, or one administrative region and to local, actionable policies. It represents clinicians’ perspective on the organizational and policy environment. The reality of preparing an interprofessional care (IPC)-ready health workforce is a complex multilayered process, starting with the beginning of education and extending to continuous professional development and the integration into the workplace structure of multiple, varied processes. This interconnection of different facilities, stakeholders, governance levels, and physical institutions demands a more systemic approach. The WHO NHWFA model allows the conceptualization of the influence of policy and law from a more managerial perspective, with attention to a range of actors, from learners to independent caregivers and systemic actors [17].

**Fig. 1:**
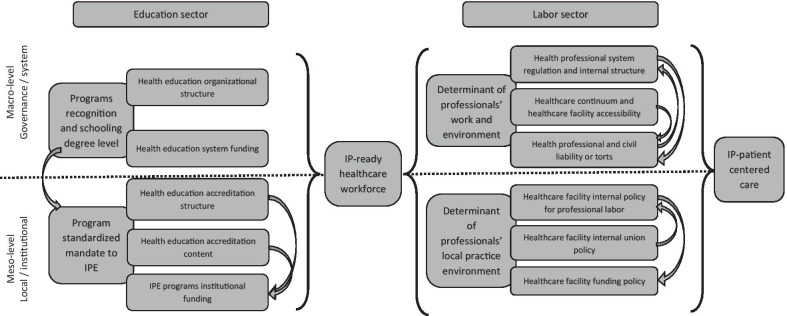
Adapted conceptual model with focus on policy and regulation aspects

For this review, we combine both models to present the literature from both the clinician perspective and the managerial perspective. The resulting conceptual model is presented in Fig. 1.

## Methods

Four databases (Medline, ProQuest, Embase, and CINAHL) were researched using the keywords “interprofessional education”, “interprofessional practice”, “collaborative practice”, “health policy”, “health law”, and “health regulation”. After the removal of duplicates, 200 abstracts were analyzed to ensure that the articles focused on policy and law. Only 34 articles dealt with the impact of policy or regulations and were retained for closer analysis. An additional 30 published items relevant to the review were found in the references. After this analysis, additional legal database research on the relevant jurisdictions’ legal environments was conducted to provide textual examples of IPECP policies or regulations.

The goal is to allow readers to reflect on their corresponding IPECP systems and identify legal or policy barriers and facilitators that can be enhanced or modified to foster IPECP. When possible, examples are provided. To ensure a common understanding of terminology, an international glossary is also provided in Additional file 1: Appendix 1.

### Education sector

The education sector is involved in interprofessional education (IPE) from the beginning of the prelicensure curriculum to the completion of the education program, when the learner enters the labor market. The impact of higher education on the achievement of healthcare innovation and desired outcomes, especially in a flexible healthcare workforce, should not be overlooked [18–20].

#### Macro-level

##### Higher education organizational structure

At the level of the higher education system, IPECP requires coordination and adaptability. A common higher education system in which each health caregiver receives and completes his or her education within a similar structure facilitates IPE promotion and implementation [21, 22]. The establishment of centralized control over the higher education process could facilitate IPE implementation by focusing the agenda on one stakeholder and emphasizing a more uniform philosophy/perception of IPECP [23, 24]. The strongest expression of centralized governmental IPE promotion would be a Ministry of Education mandate (in the form of a decree or regulation) to integrate IPE into government-approved curricula for health professionals.

An excellent example is the Danish Ministry of Education mandates on IPE. In Denmark, the government has mandated that occupational therapy, physiotherapy and nursing curricula must introduce, in practice and theory, elements of IPE [25]. The mandate does not specify the elements of accreditation per se, but programs must obey the decree to be recognized as valid by the governmental body. While there is no research specifically on the link between this policy and IPECP, there is evidence of strong collaborative patient-centered care in stroke rehabilitation driven by IP-ready professionals [26].

Although the issue of funding is beyond the scope of this article, the impact of recurrent financial support for IPE in higher education, especially when it is part of an IPE regulation or policy, should be recognized [27, 28].

#### Meso-level

##### Higher education accreditation structure

The accreditation process is a powerful instrument for implementing IPE and is usually profession-specific since the process is often linked to a regulatory body’s recognition of a program [29, 30]. A central accreditation structure can facilitate standardization if IPE/IPC is specifically introduced in accreditation criteria [23, 30–33]. Most health teaching institutions want their curricula and diplomas to be recognized as leading to licensure (a legal qualification) by the licensing body. If accreditation is required for this type of legal recognition, the presence of IPE within accreditation standards is a good foundation for IPE implementation. A regulatory body or government might choose, for the sake of worldwide recognition, to issue a policy recommendation (not an obligation) that institutions become accredited either through an international organization or a local organization using international standards. In this context, the licensing authority can still recognize all programs, even in the absence of accreditation, but professional mobility is then linked to accreditation, which can provide a strong incentive for IPE [34].

##### Higher education accreditation content

In the context of a robust accreditation system, a specific standard for IPE, regardless of the wording, underlines the importance of IPECP and has the potential to ensure consistency in the healthcare profession [30].

Two examples illustrate the IPE accreditation process. Canada’s Accreditation for IPE in Healthcare (AIPHE) project resulted in the integration of IPE language into the accreditation standards of more than 8 health professions in Canada. Starting in 2010, federal professional accreditors, with the participation of provincial colleges for each profession, mandated that IPE be an element of professional education across Canada [33]. A similar model is also present in Australia [30, 35]. These two models of profession-specific IPE accreditation standards display important diversity in both the wording and actionability of the IPE-associated standards. As underlined by a study conducted in the USA, heterogeneity in IPE accreditation standards led to a patchwork of IPC-ready professionals in the workforce but did not necessarily contribute to fostering IPC as a whole in the system [32]. Studies are currently underway to evaluate the impact of the Canadian and Australian cross-country IPE accreditation mandates.

### Labor market

Although IPE should always be present as a part of continuous professional development, the focus in the labor market is on how caregivers practice and what regulatory and legal context they practice in. Therefore, literature is on IPC more than IPE.

#### Macro-level

For labor market IPC, two aspects need to be discussed since they impact IPC in different ways: healthcare facilities and health profession policies and laws. Both aspects are macro-level aspects of the labor market, but they are often dependent on different governmental bodies or ministries. Although funding is a macro-level aspect and can impact IPC, it is beyond the scope of this article [1, 36–38].

##### Health professional system regulation and internal structure

Health professionals work in an environment that is legally governed by multiple different texts. How professions are regulated is a key aspect of professional practice. Umbrella laws, which are legal tools that regulate multiple professions under a single statute or act, are recognized as conducive to IPC [6, 7, 11, 39, 40]. Moreover, a common legal structure promotes a culture of equality among professions [36]. It also facilitates changes in professional structures, which is an important aspect of IPC [11–13, 41].

Another key aspect of professional regulation is the notion of the scope of practice, which represents the area of competence. Descriptive and adaptable scopes of practice are conducive to effective IPC because they allow health professionals to practice to the full extent of their competence without legally infringing on another profession’s scope of practice [11, 12].

Finally, an administrative structure is necessary to enforce a regulation. The existence of a global regulatory body with compulsory power (an organization with a code of ethics that regulates professional practice) can facilitate evaluation of IPC practices and the application of IPC professional obligations [28, 31, 40, 42–47].

There are multiple examples of umbrella laws across the world [40]. Structural statutes that ensure a certain uniformity within the regulation of health professions are popular [30, 35, 44, 48]. There are actually three forms of umbrella laws: the first two are top-down, with legislators giving mandates. The two models both use the descriptive scope of practice approach: the first is specific to the health and social care professions, as in Ontario, Canada [49], or Australia [35], and the second is global for all professions regardless of the activity sector, as in Quebec, Canada [44]. The latter form is bottom up: the umbrella law has the objective of fostering cooperation or collaboration among colleges, with individual statutes for every regulated profession, as in Nova Scotia, Canada [39, 50].

Since the Quebec model is the oldest in existence, there is the most evidence about its impact on IPC [51, 52], with numerous joint practice standards or guidelines from two or more professional colleges published in French on the website www.collaborationinterprofessionnelle.ca.

##### Healthcare continuum and healthcare facility accessibility

Care often occurs on a continuum. This continuum of care is anchored in structural law and policy governing whom the patient can see, where he or she can go, and who decides when and how he or she enters or leaves the premises of a healthcare institution. Often overseen by policymakers, health professional-specific control over the care continuum can favor a hidden hierarchy in IPC teams, hindering IPC efficiency [4, 36, 53–56].

There are numerous projects related to IPC and the care continuum [28, 57]. One example of the integration of IPC into care management regulations is Finland’s healthcare system. Structured around a dual-financing model of (Beveridge-type) municipal tax funding and (Bismarck-type) mandated insurance contributions, municipalities have a public mandate to organize the care continuums within their jurisdictions. This legal mandate is established by a Finnish governmental statute, the Health Care Act #1326/2010, under the Ministry of Social Affairs and Health. This 2010 statute specifically mandates that municipalities include multidisciplinary health provider teams on operating conditions (Section 4), home nursing (Section 25) and primary healthcare units (Section 35), the three main branches of the care continuum. Since 2019, these mandates have fallen under the jurisdiction of 18 newly created healthcare and welfare counties. The main disadvantages of such a primary healthcare unit system mandate are patients’ limited choice of providers [58] and the highly decentralized nature of the healthcare system, making IPC implementation difficult to study [58, 59].

##### Health professional and civil liability or torts

Although often analyzed separately, liability, or torts, is a central part of the health professional regulatory environment [12]. Independent liability can both facilitate and hinder IPC depending on the legal relationships between professions. Complete autonomy, uniform liability insurance for all and a common legal understanding are facilitators of IPC [6, 7, 60–62]. When these conditions are met, a centralized no-fault system can further mitigate the impact of liability on IPC, as seen in different countries around the globe [63, 64].

As an example, New Zealand’s no-fault compensation healthcare system was implemented in 1974. It was initially not part of the medical liability agenda but rather a result of a labor-related policy effort [63]. Since then, there have been multiple changes and additions. Putting aside the fault-driven legalistic compensation process, the New Zealand legislature chose an out-of-court approach to compensating for each “treatment injury” (articles 32 to 34 of the Accident Compensation Act 2001), regardless of the registered health providers concerned. If an injury sustained by a patient is not a normal result of the treatment or a result of an underlying disease, a structured approach to compensation is applied. Injury evaluations, compensation and claims management are performed by a public entity, namely, the Accident Compensation Corporation, which is funded by multiple public and private entities (part 7 of the Accident Compensation Act 2001). This model has the potential to limit the impact of protective behaviors on IPC.

#### Meso-level

##### Healthcare facility internal policy for professional labor

Healthcare providers act according to their locally applied policies. How these local policies are determined impacts how IPC is promoted or implemented. Thus, the commonality of professional–institutional relationships (common labor and organizational rules) in a given facility paves the way for the existence of an IPC team [14, 36, 65, 66]. Knowledge of policy content and the legal environment is key to professional engagement in IPC-promoting policy changes [67].

An example of the importance of professional engagement in policy application is the Western Cape Department of Health (WCDOH), which has an explicit policy on the importance of IPC in ensuring patient-centered care. However, this policy does not seem to have had the intended consequences. The literature seems to indicate that this policy is not embodied in the actual care model and has not broken the health profession’s hierarchical culture [68]. This example underlines the importance of clinicians’ understanding of policy content and clinicians’ knowledge of regulations and the scope of practice to ensure the success of internal policy implementation.

## Discussion and limitations

Considering the recognized importance of IPECP, the paucity of articles on related policies, regulations or legal aspects is disconcerting. Organizational models allow us to structure the available data and knowledge, but the poor legal or regulatory literacy among practicing professionals is an obstacle to the current study of IPECP policy and law [69–71]. The reflective questions presented in Table 1 have the objective of increasing readers’ awareness of the environment in their own jurisdictions.

**Table 1:** Reflective question for policy and legal environment exploration

Education sector	
Macro-level	
Higher education organizational structure	Where are health professionals trained?
	How are higher education institutions created?
	Do higher education institutions have a governmental mandate to teach IPE?
Meso-level	
Higher education accreditation structure	Do higher education healthcare programs have an obligation to be accredited either following a legal obligation or a policy recommendation?
Higher education accreditation content	If education programs/curricula are accredited, are their accreditation standards linked to IPE?
Labor market sector	
Macro-level	
Health professional system regulation and internal structure	How are health professions regulated?
	How is the scope of practice regulated?
	How are health profession regulations enforced within the professional system?
Healthcare continuum and healthcare facility accessibility	How can the patient access specialized care or in-hospital treatment?
	How is the in-hospital care episode managed?
	How is the continuum of care managed between in-hospital care and community-based care?
Health professional and civil liability or torts	How is the liability or tort system applicable to healthcare professionals or facilities?
	How is the “standard of care” determined (how is the action of one professional analyzed by judges or jurists)?
	Is there an obligation for liability insurance coverage for health professionals and/or healthcare facilities?
Meso-level	
Healthcare facility internal policy for professional labor	What is the employment relationship between healthcare employers (facilities) and healthcare professionals?
	How are care activities determined or attributed within one healthcare facility?
Healthcare facility accreditation structure and content	Is there an obligation for healthcare facilities to undergo an accreditation process before accepting patients or give care?
	Is there a specific accreditation standard or wording within different standards to mandate IPC or healthcare teams?

Often, research on policies and law are not linked to research on their effects, making such a research difficult. There is indirect evidence of the importance of policies and regulations based on either the link between policy and national culture [72] or post-policy change impact studies [14, 73]. However, we did not find evidence-based IPECP regulation or policy development (based on pre-implementation or before-and-after studies) in any published data or on any legal websites. Additionally, research on the impact of policy and law on such a complex professional practice is subject to multiple biases, especially the variable application of structural policies in practice by managers [74].

This study has several limitations. First, to avoid mistranslating policy initiatives, we limited this review to policy and laws available in English or French, thus limiting the examples to mostly developed Western countries. Second, as this research was done entirely virtually, the data used were limited to available researchable legal database without direct observation of clinicians’ behaviours.

## Conclusion

Implementing a paradigm change such as a change to IPECP is a complex endeavor. Legal aspects and policy elements contribute to this complexity. IPECP promoters and policymakers should be familiar with their local healthcare policies and legal environments to prevent inefficient policies or regulations or policy changes that do not address the broad scope of structural barriers to IPECP implementation. More research on how health professionals learn about, use and apply legal or policy elements is needed to articulate the impact of the legal environment on IPECP.

## Supplementary Information

**Additional file 1: Appendix 1.** GLOSSARY (Quotation from World Health Organization, Health Systems Strengthening Glossary, January 2011 [58]).

## Data Availability

Not applicable.

## Abbreviations

IPECP: Interprofessional education and collaborative practice
IPE: Interprofessional education
IPC: Interprofessional collaboration
WHO: World Health Organization
NHWA: National Health Workforce Account
AIPHE: Accreditation for Interprofessional Education in Healthcare
AHPRA: Australia Health Practitioner Regulatory Agency
