# Supplementary material for: Interprofessional education and collaborative practice policies and law: an international review and reflective questions
Source: Hum Resour Health. 2021 Jan 7;19:9. doi: 10.1186/s12960-020-00549-w (PMC7791710; doi:10.1186/s12960-020-00549-w)
Supplement: Supplementary file 1 — Additional file 1: Appendix 1. GLOSSARY (Quotation from World Health Organization, Health Systems Strengthening Glossary, January 2011 [58]). [file 12960_2020_549_MOESM1_ESM.docx]

APPENDIX 1: GLOSSARY (Quotation from World Health Organization, Health Systems Strengthening Glossary, January 2011(58))

| **Accreditation** | Formal process by which a recognized body, usually a non-governmental organization, assesses and recognizes that a health care organization meets applicable pre-determined and published standards.  An accreditation decision about a specific health care organization is made following a periodic on-site evaluation by a team of peer reviewers, typically conducted every two to three years.  Accreditation is often a voluntary process in which organizations choose to participate, rather than one required by law and regulation. |
| --- | --- |
| **Accreditation standards** | Accreditation standards are consensual element of evaluation, usually regarded as optimal and achievable, and are designed to encourage continuous improvement efforts within accredited organizations. |
| **Certification** | A process by which an authorized body, either a governmental or non-governmental organization, evaluates and recognizes either an individual or an organization as meeting pre-determined requirements or criteria.  Although the terms accreditation and certification are often used interchangeably, accreditation usually applies only to organizations, while certification may apply to individuals, as well as to organizations.  When applied to individual practitioners, certification usually implies that the individual has received additional education, training, and demonstrated competence in a specialty area beyond the minimum requirements set for licensure. An example of such a certification process is a physician who receives certification by a professional specialty board in the practice of obstetrics.  When applied to an organization, or part of an organization, such as the laboratory, certification usually implies that the organization has additional services, technology, or capacity beyond those found in similar organizations. |
| **Licensure** | Licensure is a process by which a governmental authority grants permission to an individual practitioner or health care organization to operate or to engage in an occupation or profession.  Licensure regulations are generally established to ensure that an organization or individual meets minimum standards to protect public health and safety.  Licensure to individuals is usually granted after some form of examination or proof of education and maybe renewed periodically through payment of a fee and/or proof of continuing education or professional competence. Organizational licensure is granted following an on-site inspection to determine if minimum health and safety standards have been met. " |
| **Regulation** | The imposition of external constraints upon the behaviour of an individual or an organization to force a change from preferred or spontaneous behaviour. |
| **Regulations** | Rules and administrative codes issued by governmental agencies at all levels, municipal, county, state and federal. Although they are not laws, regulations have the force of law, since they are adopted under authority granted by statutes, and often include penalties for violations |
| **Statutes** | Federal or state written law enacted by the Congress or state legislature, respectively. Local statutes or laws are usually called "ordinances." Regulations, rulings, opinions, executive orders and proclamations are not statutes. |
